# The possible cardioprotective effect of ghrelin during experimental endotoxemia in mice

**DOI:** 10.25122/jml-2023-0228

**Published:** 2024-05

**Authors:** Zinah Majid, Bashaer Muhammad-Baqir, Dhirgam Falih Al-Shimerty, Najah Rayish Hadi

**Affiliations:** 1 Southern Primary Health Sector, Najaf, Iraq; 2 Department of Clinical Pharmacy, Faculty of Pharmacy, University of Kufa, Kufa, Iraq; 3 Department of Pharmacology and Toxicology, Faculty of Pharmacy, University of Kufa, Kufa, Iraq; 4 Department of Pharmacology & Therapeutics, Faculty of Medicine, University of Kufa, Kufa, Iraq

**Keywords:** ghrelin, sepsis, CLP, cardiac, TNF-α, MIF, TLR4, 8-epi-PGF2α

## Abstract

This study aimed to evaluate the cardioprotective effects of ghrelin in septic mice, focusing on its anti-inflammatory and antioxidant properties. Thirty-five male Swiss mice (8-12 weeks old, 23–33g) were randomly assigned to five groups (*n* = 7 each): (1) Normal, fed usual diets, (2) Sham, subjected to anesthesia and laparotomy, (3) Sepsis, subjected to cecal ligation and puncture, (4) Vehicle, given an equivalent volume of intraperitoneal saline injections immediately after cecal ligation and puncture, and (5) Ghrelin-treated, administered 80 µg/kg ghrelin intraperitoneal injections immediately following cecal ligation and puncture. Serum levels of tumor necrosis factor-alpha (TNF-α), macrophage migration inhibitory factor (MIF), toll-like receptor 4 (TLR4), and 8-epi-prostaglandin F2 alpha (8-epi-PGF2α) were measured. The extent of cardiac damage was also evaluated histologically. The mean serum levels of TNF-α, MIF, TLR4, and 8-epi-PGF2α levels were significantly higher in the sepsis and vehicle groups than in the normal and sham groups. The levels were significantly lower in the ghrelin-treated group than in the vehicle and sepsis groups. Histological analysis revealed normal myocardial architecture in the normal and sham groups, whereas the sepsis and vehicle groups had severe myocardial injury. The ghrelin-treated group displayed histological features similar to the sham group, indicating reduced myocardial damage. Ghrelin ameliorated sepsis-induced cardiotoxicity in mice by exhibiting strong anti-inflammatory and antioxidant effects. These findings suggest that ghrelin may be a promising therapeutic candidate for the prevention of sepsis-induced cardiotoxicity.

## INTRODUCTION

Sepsis, a complex clinical syndrome characterized by life-threatening organ dysfunction due to a dysregulated host response to infection, is associated with significant mortality and morbidity. Excessive production of proinflammatory cytokines by activated immune cells can lead to multiple organ failure and death [1]. According to the Centers for Disease Control and Prevention (CDC), sepsis is responsible for one-third of all hospital deaths in the United States. It is estimated that 1.7 million Americans are diagnosed with sepsis each year, with approximately 270,000 of these cases resulting in mortality. Sepsis management is also the most expensive medical condition in US hospitals, costing over $24 billion annually [2].

Septic cardiomyopathy, a frequent and severe consequence of sepsis, is characterized by ventricular hypertrophy and impaired contractility, leading to increased hospital stays, mortality rate, and cardiovascular (CV) morbidity risk after discharge [3,4]. Nevertheless, there is currently no recognized therapeutic strategy for ameliorating septic cardiomyopathy. Ghrelin is a polypeptide hormone comprising 28 amino acids, primarily released in the stomach. The primary physiological role of ghrelin is to induce growth hormone (GH) secretion from the pituitary gland, and this effect is dose-dependent [5]. Beyond its endocrine effects on GH, ghrelin also influences the release of gonadotropin, prolactin, and cortisol [6] and plays a crucial role in maintaining positive energy balance and promoting fat deposition [7]. Numerous studies demonstrated that some mammalian peptide hormones have promising effects against sepsis, including vasopressin, oxytocin, human chorionic gonadotropin, ghrelin, and glucagon [8]. Ghrelin exhibits potent anti-inflammatory properties, suppressing the expression of proinflammatory cytokines such as tumor necrosis factor-alpha (TNF-α), interleukin (IL)-6, and IL-1β in T lymphocytes, macrophages, and monocytes [9]. In addition, it enhances the synthesis of IL-10, an anti-inflammatory cytokine [10]. Additionally, ghrelin administration inhibits reactive oxygen species (ROS) formation and elevated antioxidant enzyme levels, such as superoxide dismutase or glutathione. In addition to its endocrine and immunomodulatory actions, ghrelin exerts diverse effects on the cardiovascular (CV) system in both physiological and pathological states. These effects include modulation of vascular tone, cardiac function, and blood volume regulation [11]. Emerging evidence suggests ghrelin may offer cardioprotection by mitigating thrombosis, stabilizing atherosclerotic plaques, and attenuating inflammation [12]. In the context of sepsis, ghrelin administration has been shown to improve myocardial contractility and ventricular peak systolic pressure, potentially through direct actions on the ventricles and by reducing the levels of proinflammatory cytokines like TNF-α [13]. TNF-α is a proinflammatory cytokine released from immune cells such as T lymphocytes and non-immune cells such as fibroblasts [14]. During sepsis, elevated blood and myocardial levels of TNF-α are related to both functional and cellular cardiac impairment [15]. The macrophage migration inhibitory factor (MIF), an essential proinflammatory cytokine, is recognized to be involved in inflammatory CV diseases, such as myocarditis [16] and septic cardiomyopathy [17]. Toll-like receptors (TLRs) are innate immune receptors that recognize various pathogen-associated molecular patterns derived from pathogens [18] and damage-associated molecular patterns released from injured tissue [19]. TLRs upregulated the production of inflammatory cytokines via multiple signaling pathways. F2-isoprostanes are prostaglandin-like compounds produced when polyunsaturated arachidonic acid undergoes peroxidation in the presence of free radicals [20]. 8-iso-Prostaglandin F2α (8-iso-PGF2α) is the most measured biomarker for quantifying F2-isoprostanes, which are reliable indicators of oxidative stress [21].

This study aimed to evaluate the cardioprotective effect of ghrelin in septic mice, focusing on its anti-inflammatory and antioxidant properties.

## MATERIAL AND METHODS

Thirty-five adult male albino Swiss mice weighing 23-33g, aged 8-12 weeks, were obtained from the Iraqi Center for Cancer Research and housed in the animal facility at the Faculty of Pharmacy/University of Kufa. They were housed in cages with a 12-hour light/12-hour dark cycle, with temperatures ranging from 22-24 °C, humidity levels ranging from 60-65%, and unrestricted access to water and food. The study was conducted in the laboratory of the Clinical Laboratory Department, Faculty of Pharmacy, University of Kufa, from December 5, 2022, to February 25, 2023.

### Study design

Following a one-week acclimatization period, the mice were randomly assigned to five groups (*n* = 7 per group):

1. Normal group: Mice were fed their usual diets until the time of sampling.
2. Sham group: Mice were subjected to anesthesia and laparotomy, serving as the negative surgical control group.
3. Sepsis group: Mice underwent a cecal ligation and puncture (CLP) procedure, serving as the positive surgical control group.
4. Vehicle group: Mice received an equivalent volume of normal saline intraperitoneal injections immediately following CLP.
5. Ghrelin-treated group: Mice were administered 80 µg/kg of recombinant human ghrelin intraperitoneal injections immediately following CLP.

Twenty hours after CLP [22], mice were euthanized, and myocardial tissue and serum samples were collected.

### Experimental model of sepsis

Polymicrobial sepsis was induced using the CLP model [23,24]. Mice were anesthetized with intraperitoneal injections of xylazine (0.01 mg/g) and ketamine (0.1 mg/g). A 1.5 cm midline abdominal incision was made, and the cecum was ligated below the Bauhin valve, then perforated twice with a 22-gauge needle. The cecum was gently squeezed to extrude a small amount of stool and returned to its anatomical position. The abdominal incision was then closed with a 4-3 surgical suture.

### Preparation of ghrelin

Recombinant pure human ghrelin (95%, Elabscience) was dissolved in normal saline. An 80 µg/kg dose of ghrelin was administered intraperitoneally immediately after CLP [25].

### Sample collection

#### Blood samples

Blood samples were collected by heart puncture before euthanizing mice. The samples were placed in gel tubes and left at room temperature for one hour. The serum was separated by centrifugation at 4000 rpm for 20 minutes. Serum levels of TNF-α, macrophage migration inhibitory factor (MIF), toll-like receptor 4 (TLR4), and 8-epi-prostaglandin F2 alpha (8-epi-PGF2α) were measured using enzyme-linked immunosorbent assay (ELISA).

#### Tissue samples

Cardiac tissue was fixed in a 10% formaldehyde solution for 20 hours. After dehydration and clearing, the cardiac tissue was embedded in paraffin blocks, and 5 µm thick sections were sliced using a microtome. Hematoxylin and eosin (H&E) staining was performed for histological examination under a light microscope [26].

### Histological examination

Cardiac damage was evaluated using an optical microscope, and photographs were taken of the sections. Histological sections were scored based on the Zingarelli protocol. The criteria for this scoring system were:

- Score 0: No damage.
- Score 1: Localized necrosis with interstitial edema.
- Score 2: Diffuse swelling of cardiomyocytes.
- Score 3: Leukocyte infiltration and contraction band.
- Score 4: Contraction band, neutrophil infiltration, and hemorrhage.

### Statistical analysis

Statistical analysis was performed using GraphPad Prism version 8.1. Data were expressed as mean ± standard error of the mean (SEM). Group comparisons were made using one-way analysis of variance (ANOVA), followed by Bonferroni post-hoc tests for multiple comparisons. Histopathological changes were compared using a non-parametric test and Dunn's post-hoc test. A *P* value of < 0.05 was considered statistically significant.

## RESULTS

### Effect of ghrelin treatment on TNF-α after polymicrobial sepsis

**Figure 1:**
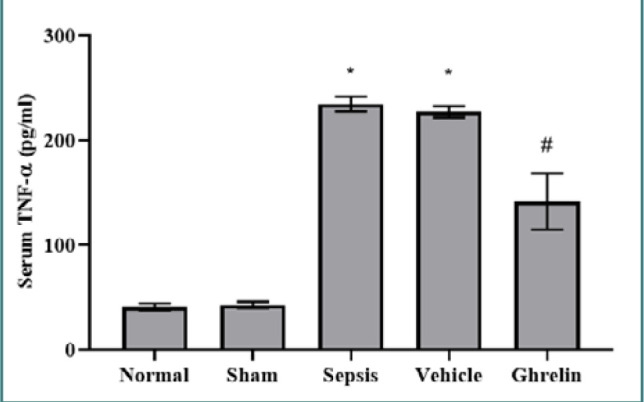
Serum TNF-α levels in the experimental groups *Significant *P* <0.001 vs. normal or sham groups; #Significant *P* <0.001 vs. sepsis or vehicle groups.

Serum levels of TNF-α were significantly higher in the sepsis group than in the normal and sham groups (*P* < 0.001). There were no statistically significant variations in serum TNF-α levels between the vehicle and sepsis groups or between the normal and sham groups. Compared to both the vehicle and sepsis groups, a single dose of ghrelin administered immediately after the CLP procedure significantly (*P* <0.001) lowered serum TNF-α levels (Figure 1).

### Effect of ghrelin treatment on MIF after polymicrobial sepsis

**Figure 2:**
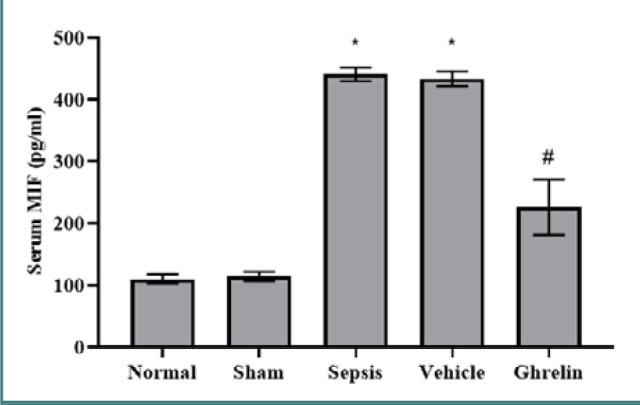
Serum MIF level in the experimental groups *Significant (P <0.001) vs. normal or sham groups; #Significant (P <0.001) vs. sepsis or vehicle groups.

Serum MIF levels were significantly higher in the sepsis group than in the normal and sham groups (*P* <0.001). There were no statistically significant variations in serum MIF levels between the vehicle and sepsis groups or between the normal and sham groups. Compared to both the vehicle and sepsis groups, a single dose of ghrelin administered immediately after the CLP procedure significantly lowered serum MIF levels (*P* <0.001) (Figure 2).

### Effect of ghrelin treatment on TLR4 after polymicrobial sepsis

**Figure 3:**
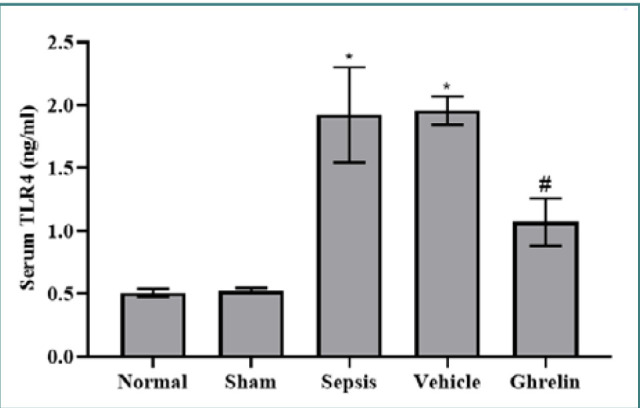
Serum TLR4 level in the experimental groups *Significant *P* <0.001 vs. normal or sham groups; #Significant *P* <0.001 vs. sepsis or vehicle groups.

Serum TLR4 levels were significantly higher in the sepsis group compared to the normal and sham groups (*P* <0.001). There were no statistically significant differences in serum TLR4 levels between the vehicle and sepsis groups or between the normal and sham groups. Compared to both the vehicle and sepsis groups, a single dose of ghrelin administered immediately after the CLP procedure significantly lowered serum TLR4 levels (*P* <0.001) (Figure 3).

### Effect of ghrelin treatment on 8-epi-PGF2α after polymicrobial sepsis

**Figure 4:**
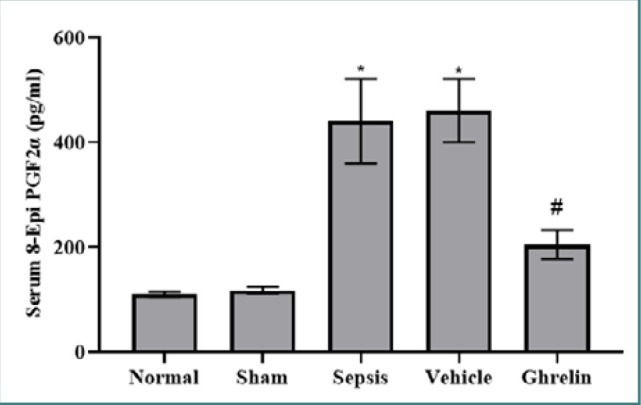
Serum 8-Epi PGF2α level in the experimental groups *Significant *P* <0.001 vs. normal or sham groups; #Significant *P* <0.001 vs. sepsis or vehicle groups.

Serum 8-epi-PGF2α levels were significantly higher in the sepsis group than in the normal and sham groups (*P* <0.001) (Figure 4).

There were no statistically significant variations in serum 8-epi-PGF2α levels between the vehicle and sepsis groups or between the normal and sham groups. In comparison to both the vehicle and sepsis groups, a single dose of ghrelin administered immediately after the CLP procedure significantly lowered serum 8-epi-PGF2α levels (*P* <0.001) (Figure 4).

### Histopathological changes of myocardial tissue after polymicrobial sepsis

**Figure 5:**
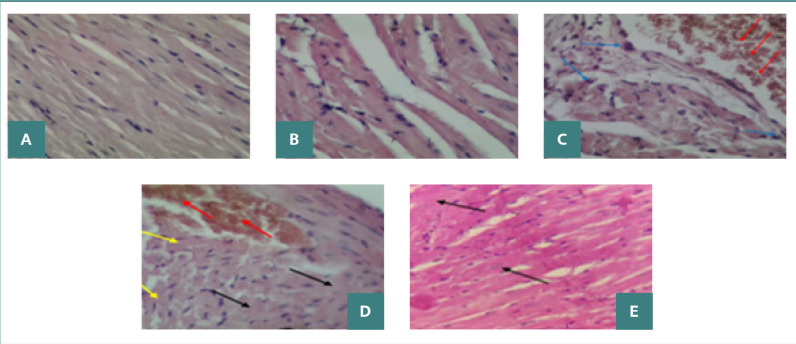
Photograph of heart sections stained with H&E (X400). A, normal group, showed normal architecture (score 0); B, the sham group, showed normal architecture (score 0); C, sepsis group, showed hemorrhage (red arrows) and leukocyte infiltration (blue arrows); D, vehicle group showed hemorrhage (red arrows), cell swelling (yellow arrows), and necrosis (black arrows); E, ghrelin group showed mild necrosis (black arrows).

Normal and sham groups myocardium had normal architecture with distinct myocyte boundaries and without erythrocyte leakage and leukocyte infiltration (Figure 5 AB). All mice in these groups had normal histopathological findings (score 0). The sepsis and vehicle groups had a highly severe myocardial injury (score 4), characterized by the appearance of contraction bands, interstitial edema, leukocyte infiltration, and erythrocyte extravasation (Figure 5 CD). The mean histological score was significantly higher in the sepsis and vehicle groups than in the normal and sham groups (*P* <0.001).

**Figure 6:**
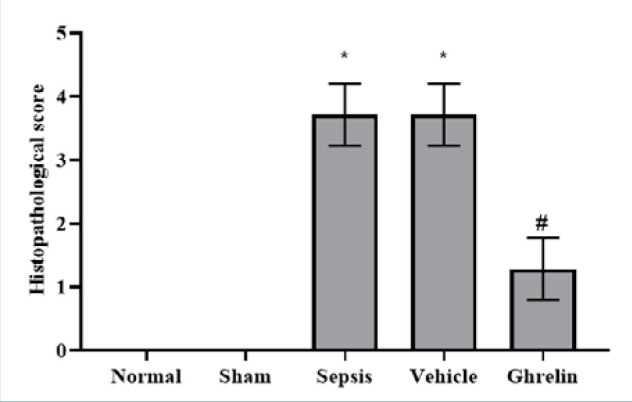
Histopathological score in the experimental groups *Significant (P <0.001) vs. normal or sham groups; #Significant (P <0.001) vs. sepsis or vehicle groups.

The ghrelin-treated group had a mild myocardial injury (score 1) (Figure 5 E). The mean histological score was significantly lower in the ghrelin-treated group than in the sepsis and vehicle groups (*P* <0.01) (Figure 6).

## DISCUSSION

Sepsis is a life-threatening condition characterized by vital organ dysfunction resulting from an uncontrolled host response to infection [1]. Various pathogens, including bacteria, parasites, fungi, and viruses, can precipitate sepsis [27,28]. In this study, serum TNF-α levels were significantly higher in the sepsis and vehicle groups than in the normal and sham groups. However, the administration of ghrelin immediately after induction of sepsis significantly lowered TNF-α levels compared to sepsis and vehicle groups. Senousy *et al*. [29] showed that CLP-induced sepsis significantly increased serum TNF-α levels compared to the sham group. Furthermore, Lee *et al*. [30] demonstrated that TNF-α serum levels increased significantly at 4 and 6 hours after CLP-induced sepsis compared to the sham-operated rats. Conversely, Cheng *et al*. [31] revealed that ghrelin significantly reduced serum TNF-α levels in mice 72 hours after intracranial hemorrhage. Another study showed that cardiac tissue TNF-α and IL-6 levels were significantly lower in the ghrelin-treated group compared to the sepsis model group [32]. Moreover, El-Shaer *et al*. [33] demonstrated that exogenous ghrelin administration had cardioprotective effects in rats with isoproterenol-induced myocardial ischemia by reducing serum and cardiac tissues levels of oxidative stress and proinflammatory mediators, including TNF- α and IL-6 [34]. These findings align with our study, underscoring the cardioprotective effects of ghrelin by reducing proinflammatory cytokines.

Our study also found significantly higher serum MIF levels in the sepsis and vehicle groups compared to the normal and sham groups. Conversely, the ghrelin-treated group had significantly lower MIF serum levels than the sepsis and vehicle groups. Li *et al*. [30] reported elevated MIF levels in CLP-operated mice than in sham-operated mice. Another study revealed that sepsis increased MIF levels in lung tissue relative to the sham group [35]. Gonzalez-Rey *et al*. [36] found that ghrelin administration significantly decreased MIF mRNA and protein expression in mucosal immune cells in a mouse model of colitis. To our knowledge, no previous study has specifically examined the effects of ghrelin on MIF in polymicrobial sepsis. Our findings suggest that ghrelin effectively reduced MIF levels, contributing to its cardioprotective effects.

Serum TLR4 levels were also significantly elevated in the sepsis and vehicle groups compared to the normal and sham groups. In contrast, the ghrelin-treated group had significantly lower serum levels of TLR4 compared to the vehicle and sepsis group. Yildiz *et al*. [37] found that TLR4 expression was higher in the lung tissue of septic rats than in the sham group. Several studies confirmed an association between ROS and the incidence and progression of sepsis-induced organ dysfunction. ROS production during sepsis promotes cardiomyocyte damage, leading to septic cardiomyopathy. Alnfakh *et al*. [24] revealed that sepsis increased lung tissue 8-epi-PGF2α levels relative to the sham group. Another study showed that lung tissue 8-epi-PGF2α levels in the CLP-operated mice were significantly increased compared to the sham-operated mice [35]. To our knowledge, no previous study has shown the impacts of ghrelin on 8-epi-PGF2α in polymicrobial sepsis.

Histopathological examination revealed severe myocardial injury in the sepsis and vehicle groups, characterized by contraction bands in the myofibrils, blood cell extravasation, cellular swelling, necrosis, karyolysis, pyknosis, and karyorrhexis. The upregulation of inflammatory cytokines and the elevation of oxidant molecule levels in the heart during polymicrobial sepsis account for these histological alterations. The present study showed that sepsis and vehicle groups had significantly higher cardiac tissue injury than normal and sham groups. The histopathological damage scores were mostly highly severe (score 4) for sepsis and vehicle groups. However, ghrelin administration significantly reduced myocardial tissue injury compared to vehicle and sepsis groups. The ghrelin-treated group had mild (score 1) histopathological damage scores. Our findings confirm the cardioprotective role of ghrelin by reducing proinflammatory cytokines, including TNF-α, MIF, and TLR4, and the oxidative stress marker, 8-epi-PGF2α. Zigam *et al*. [25] found that the cardiac tissue in the septic group was seriously damaged and characterized by leukocyte infiltration, contraction bands, interstitial edema, internal bleeding, and necrosis. In contrast, Wang *et al*. [12] found that ghrelin effectively reduced myocardial injury in the ischemia/reperfusion rat model, evidenced by decreased cardiomyocyte apoptosis, infarct size, and cardiac injury biomarkers. Moreover, the injection of ghrelin relieved inflammatory response and oxidative stress following ischemia/reperfusion injury. El-Shaer *et al*. [33] reported that the antioxidant and anti-inflammatory properties of ghrelin are responsible for its cardioprotective effects in the rat model of isoproterenol-induced myocardial injury.

This study has several limitations that should be acknowledged. The sample size was relatively small, which may affect the generalizability of the findings. Additionally, we did not measure cardiac biomarkers and hemodynamic parameters, which could provide further insights into the cardioprotective effects of ghrelin. The effects of ghrelin administration were conducted at a single time point, limiting our understanding of its long-term impact. Moreover, further studies are necessary to investigate the underlying mechanisms responsible for the observed cardioprotective effects of ghrelin. These limitations suggest the need for more comprehensive research to fully elucidate the therapeutic potential of ghrelin in sepsis-induced cardiotoxicity.

## CONCLUSION

Our study demonstrated that ghrelin had significant cardioprotective effects in a mouse model of polymicrobial sepsis. The administration of ghrelin resulted in a significant reduction in proinflammatory cytokines, including TNF-α, MIF, and TLR4, and decreased levels of the oxidative stress marker 8-epi-PGF2α. Histopathological examination of myocardial tissue further confirmed the protective role of ghrelin, showing significant improvements in tissue architecture and reduced injury scores. These results indicate that ghrelin exerts its cardioprotective effects through potent anti-inflammatory and antioxidant mechanisms, as well as by mitigating histopathological damage. Given these findings, ghrelin holds promise as a therapeutic candidate for the prevention and treatment of sepsis-induced cardiotoxicity.
